# An Examination of the Association of Selected Toxic Metals with Total and Central Obesity Indices: NHANES 99-02

**DOI:** 10.3390/ijerph7093332

**Published:** 2010-08-26

**Authors:** Miguel A. Padilla, Mai Elobeid, Douglas M. Ruden, David B. Allison

**Affiliations:** 1 Department of Psychology, Old Dominion University, 250 Mills Godwin Building, Norfolk, VA 23529, USA; E-Mail: mapadill@odu.edu; 2 Department of Mathematics and Statistics, Old Dominion University, 250 Mills Godwin Building, Norfolk, VA 23529, USA; 3 Department of Biostatistics, University of Alabama at Birmingham, 1665 University Boulevard, Birmingham, AL 35294, USA; E-Mail: maielobeid@gmail.com; 4 Division of Cardiovascular Disease, Department of Medicine, University of Alabama at Birmingham, 1665 University Boulevard, Birmingham, AL 35294, USA; 5 Institute of Environmental Health Sciences, Wayne State University, 259 Mack Avenue, Detroit, Michigan 48201, USA; E-Mail: douglasr@wayne.edu; 6 Department of Obstetrics and Gynecology, Wayne State University, Wayne State University, 275 E. Hancock Avenue, Detroit, MI 48201, USA; 7 Department of Nutrition Sciences, University of Alabama at Birmingham, 1665 University Boulevard, Birmingham, AL 35294, USA; 8 Clinical Nutrition Research Center, University of Alabama at Birmingham, 1665 University Boulevard, Birmingham, AL 35294, USA

**Keywords:** obesity, endocrine disruptors, waist circumference, toxic metals, public health

## Abstract

It is conceivable that toxic metals contribute to obesity by influencing various aspects of metabolism, such as by substituting for essential micronutrients and vital metals, or by inducing oxidative stress. Deficiency of the essential metal zinc decreases adiposity in humans and rodent models, whereas deficiencies of chromium, copper, iron, and magnesium increases adiposity. This study utilized the NHANES 99-02 data to explore the association between waist circumference and body mass index with the body burdens of selected toxic metals (barium, cadmium, cobalt, cesium, molybdenum, lead, antimony, thallium, and tungsten). Some of the associations were significant direct relationships (barium and thallium), and some of the associations were significant inverse relationships (cadmium, cobalt, cesium, and lead). Molybdenum, antimony, and tungsten had mostly insignificant associations with waist circumference and body mass index. This is novel result for most of the toxic metals studied, and a surprising result for lead because high stored lead levels have been shown to correlate with higher rates of diabetes, and obesity may be a key risk factor for developing diabetes. These associations suggest the possibility that environmental exposure to metals may contribute to variations in human weight gain/loss. Future research, such as prospective studies rather than the cross-sectional studies presented here, is warranted to confirm these findings.

## 1. Introduction

The marked increases in prevalence of overweight and obesity over the last several decades in the United States poses a major public health concern [1]. Toxic metals may be conjectured to play a role in contributing to obesity by displacing vital metals such as zinc, chromium, copper, iron, and magnesium, which may in turn affect energy production, carbohydrate tolerance, and other metabolic process[2]. In mice, zinc deficiency induced by a mutation in the Znt7 zinc transporter caused a reduced body weight gain that was largely due to the decrease in body fat accumulation [3]. In contrast, deficiency of other essential metals, chromium [4,5], copper [6], iron [7,8], and magnesium [9], causes an increase in adiposity. The toxic metals, lead and cadmium are ubiquitous environmental toxins that are related to a broad range of physiologic, biochemical, and behavioral dysfunctions[10,11] and recent epidemiologic studies have reported that environmental exposure to lead or cadmium concentration has a graded association with several disease outcomes such as hypertension, peripheral artery diseases, kidney diseases, and cognitive impairment [12–17]. To our knowledge, this is the first study to demonstrate an association between lead and other toxic metals and obesity.

Heavy metals are a heterogeneous group of highly reactive substances, which may act as essential cofactors for physiologic processes and/or as toxic elements. Some metals induce oxidative stress directly, such as iron redox-cycling between Fe^3+^ and Fe^2+^ and creating superoxide (O_2_^−^) in the process [18]. Whereas iron (Fe), copper (Cu), chromium (Cr), vanadium (V) and cobalt (Co) undergo redox-cycling reactions under physiological conditions, other metals such as mercury (Hg), cadmium (Cd) and nickel (Ni), and arsenic (As) increase oxidative stress indirectly by depleting glutathione and bonding to sulfhydryl groups of proteins [18]. The unifying factor in determining metal toxicity is the generation of reactive oxygen and nitrogen species [18].

The toxic metals we investigated in this study range from ubiquitous to rare in the environment. Lead, cadmium, cobalt, antimony, and cesium are all on the 2007 CERCLA (Comprehensive Environmental Response, Compensation, and Liability Act) priority list of hazardous substances (http://www.atsdr.cdc.gov/cercla/index.html). The CERCLA requires the EPA to prepare a list, in order of priority, of substances that are most commonly found at Superfund sites (also known as the National Priority List (NPL) sites). These contaminants are determined to pose the most significant potential threat to human health due to their known or suspected toxicity and potential for human exposure at these NPL sites.

As an initial exploratory inquiry, the present study investigated the association of body mass index (BMI) and waist circumference (WC) with toxic metals adjusting for age, ethnicity, and gender via multiple linear regressions using participants aged 6–60 across National Health and Nutrition Examination Survey (NHANES) 99-02. BMI is calculated as weight in kilograms/height in meters squared and WC is measured in centimeters. The toxic metals examined included barium, cadmium, cobalt, cesium, molybdenum, lead, antimony, thallium, and tungsten.

## 2. Experimental Section

### 2.1. Samples

We used data from the publicly available National Health and Nutrition Examination Survey (NHANES) from 1999–2000 to 2001–2002. Collected and maintained by the US National Center for Health and Statistics (NCHS), the data are from a cross-sectional survey designed to yield a representative sample of the U.S. non-institutionalized civilian population. The surveys have the same basic structure and plan, and contain age, gender, race, height, weight, and WC. Anthropometric measures including height, weight, and WC were also obtained via the NHANES database (http://www.cdc.gov/nchs/data/nhanes/frequency/bmxdoc.pdf). In addition, the surveys are stratified multistage probability samples based on selection of counties, blocks house-holds, and persons within households.

Measures of metals were assessed in urine samples from one-third of participants. For this analysis, data from two NHANES surveys were aggregated (1999–2000, 2001–2002). Metals were all measured as individual chemicals by high resolution gas chromatography mass spectrometry (GC/MS) using isotope dilution for quantification. The metals data are adjusted for serum total cholesterol and triglycerides. In addition, the models were adjusted for the following two variables: socioeconomic status and Creatinine. Socioeconomic status is adjusted for by including the poverty income ratio (PIR). NHANES indicates that PIR < 1 are below the official poverty threshold and PIR ≥ 1 are above the poverty level. Creatinine, a break down product of creatine phosphate, was used as a controlling covariate because some heavy metals such as lead damage the kidneys and consequently cause an increase in serum creatinine, which is normally filtered by the kidneys [19]; creatinine levels are therefore relative measures of kidney damage. All metals levels were transformed by taking the natural log (ln) of each in order to improve linearity and analyzed in their transformed form.

Based on relatively high prevalence in the NHANES population, which is needed for statistical analyses, we selected nine metals: barium, cadmium, cobalt, cesium, molybdenum, lead, antimony, thallium, and tungsten. Based on the above criteria, a total of approximately 3,816 participants with valid BMI scores and 3,825 participants with valid WC score were used.

### 2.2. Statistical Analysis

The primary analysis consisted of three regression models using all available participants testing for associations with adiposity as assessed by BMI and WC. Two models regressed BMI and WC respectively on gender, ethnicity, PIR, age, creatinine, and metals. All variables are included in the model simultaneously with the first five variables acting as covariates. The third model regressed WC on the previous predictors but controlled for BMI. These models are referred to as additive models. Each of these models was extended by including two-way interactions between gender and metals.

All analysis, including descriptive statistics, were conducted using SUDAAN 9.01 (RTI International, Research Triangle Park, NC) or AM Statistical Software v.0.06 (The American Institutes for Research, DC), which estimates standard errors using the sampling weights, strata, and primary sampling units (PSU) from NHANES allowing for the complex sampling procedures used.

## 3. Results and Discussion

### 3.1. Descriptive Statistics

Descriptive statistics for BMI and WC by gender, ethnicity, and age are displayed in Table 1.

Correlations between BMI and WC with all variables in the models are displayed in Table 2.

Inspection of the quantities of the correlation matrix indicates that multi-collinearity is not a major problem. The highest correlations are between creatinine and the metals, but creatinine is being used as a control variable in the models to take into account the toxic effect of metals on creatinine on the kidneys. After creatinine, the correlation between the metals is the next highest group. This might be caused by a relatively high rate of co-exposures to these metals, such as via proximity to industrial waste or incinerators.

### 3.2. Additive Models

Results of the additive regression models for all participants are presented in Table 3 with corresponding least square means in Table 4.

In the model testing for the association with BMI, there are significant associations, independent of the covariates, for ln barium, ln cadmium, ln cobalt, ln cesium, ln lead, and ln thallium, with the model accounted for approximately 28% of the BMI variance (Table 3), with 3% of the variance uniquely attributable to the metals. The associations are positive for ln barium and ln thallium. The significant metal dose-responses for BMI are presented in Table 5. On the other hand, the associations are negative for ln cadmium, ln cobalt, ln cesium, and ln lead. Age has a curvilinear association with BMI in that BMI increases at early ages, peaks around midlife, and begins decreasing.

For testing the association with WC, there are significant associations, independent of the covariates, for ln barium, ln cadmium, ln cobalt, ln cesium, ln lead, and ln thallium. The model accounted for approximately 40% of the WC variance (Table 3), with 3% of the variance uniquely attributable to the metals. The associations are positive for ln barium and ln thallium, yet negative for ln cadmium, ln cobalt, ln cesium, and ln lead. The significant metal dose-responses for WC are presented in Table 6. As in the previous molded for BMI, age has a curvilinear association with WC in that WC increases at early ages, peaks around midlife, and then begins decreasing.

Because of the high association of creatinine with obesity, a second set of regression models were estimated without using creatinine as a covariate. Results of these models are presented in Table 7. The analyses largely indicate that the majority of results remain unchanged. The exceptions were the parameters estimates for ln molybdenum and ln tungsten which change direction without creatinine in the model. However, neither ln molybdenum nor ln tungsten are significant whether creatinine is used as a covariate or not. Additionally, ln antimony became significant when creatinine was not used as a covariate. Even so, the results remain largely unchanged whether creatinine is used as a covariate or not.

Lead remained clearly consistent whether creatinine is used as a covariate or not. In order to see the association of lead with BMI and WC, a regression model was estimated using only ethnicity, gender, and age as covariates. Table 8 displays these results. The parameter for ln lead remained in the same direction, but it is no longer significant. So it appears that all the metals must be taken into account in order to see the true association of ln lead with BMI and WC.

For testing the effects on WC conditional on BMI, there were no significant main associations for any of the metals controlling for the covariates. Evidence suggests that WC is increasing in the population and is considered as a marker for body fat distribution [20–25], hence the analysis was included.

### 3.3. Interaction Models

Although the joint test for the interaction between gender and all of the metals were significant for both the BMI and WC models, the change in variance seemed trivial. Specifically, the incremental variance due to the interactions was always less than 0.05%. For this reason, the interaction models were not pursued.

### 3.4. Models for Adolescents and Adults

Because of the rapid changes of BMI from ages 6–18, the same analyses were conducted for individuals ages 6–18 (adolescents) or 19 or older (adult). Tables 9 to 14 present the results of these analyses. For adolescents, only barium, cobalt, and lead remained consistently significant. The significant metal dose–responses for BMI and WC are presented in Tables 10 and 11, respectively. For adults, with the exception of cobalt, all of the parameters estimates remained in the same direction and those that were significant in the original model remained significant. The significant metal dose-responses for BMI and WC are presented in Tables 13 and 14, respectively.

## 4. Conclusions

Our results indicate several significant associations. The toxic metals barium and thallium are positively associated with BMI and WC, whereas cadmium, cobalt, cesium, and lead are negatively associated with BMI and WC. Most toxic metals such as cadmium have more than one oxidation state; accordingly the same metal may have different effects on humans [18]. Weight gain or weight loss, depending on the toxic metal, tends to occur at much lower levels of exposure of metals than those that make animals or humans obviously ill. For example, the CDC health concern blood lead level (BLL) is 10 μg/dL, and higher levels warrant intervention such as chelation therapy, especially in children [26]. The mean BLL in the NHANES, 1999–2000, population is 2.7 μg/dL and only 4.4% had BLL concentrations above the CDC health concern level [27]. A broad range of lead levels had negative associations with BMI and WC, suggesting that levels much lower than 10 μg/dL affect these traits. This result is consistent with studies of neurodevelopmenttal defects in children associated with low blood lead levels that have led to call for the Centers for Disease Control and Prevention to reduce the current screening guideline of 10 μg/dL [28–31]. Exposure to lead can happen from breathing workplace air or dust, eating contaminated foods, or drinking contaminated water. Lead has been found in at least 1,272 of the 1,684 National Priority List (NPL) sites identified by the Environmental Protection Agency (EPA) (http://www.atsdr.cdc.gov/cercla/index.html).

The other toxic metals in this study have various sources. Exposure to barium occurs mostly in the workplace or from drinking contaminated water. Barium and barium compounds have been found in at least 798 of the 1,684 NPL sites identified by the EPA. Exposure to thallium occurs mainly from eating food, but high levels of exposure may occur in the workplace. Thallium has been found in at least 210 of 1,416 NPL sites identified by the EPA. Exposure to cadmium happens mostly in the workplace, but lower doses are obtained from breathing cigarette smoke or eating cadmium contaminated foods. Cadmium has been found in at least 1,014 of the 1,669 NPL sites identified by the EPA. The general population is exposed to low levels of cobalt in air, water, and food. Cobalt has both beneficial effects, as part of vitamin B12, and harmful effects on health at high doses. Cobalt has been found in at least 426 of the 1,636 NPL sites identified by the EPA. Exposure to stable or radioactive cesium occurs from ingesting contaminated food or drinking water or breathing contaminated air. High levels of radioactive cesium may occur after nuclear accidents or detonation of atomic bombs. Stable (non-radioactive) cesium has been found in at least 8 of the 1,636 NPL sites and radioactive cesium has been found in at least 23 of the 1,636 NPL sites identified by the EPA.

One possible explanation for why some toxic metals (barium and thallium) positively associate with obesity is that they induce oxidative stress, which increases lipogenesis at the expense of energy production [32]. Oxidative stress can be either by directly generating free radicals, in the case of redox-cycling metals such as barium or thallium, or indirectly by non-redox cycling metals such as lead and mercury [18]. Indirect induction of oxidative stress can be mediated by reducing glutathione levels or by interfering with iron metabolism. Reactive oxygen species directly or indirectly generated by metals can inhibit the normal mitochondrial metabolic function, and prevent the mitochondria from producing energy, in the form of ATP, by oxidative phosphorylation. The lower levels of ATP, coupled by the diminished efficacy of the tricarboxylic acid (TCA) cycle due to inhibition of enzymes such as aconitase, which are sensitve to oxidative stress, would cause the liver to divert metabolites to lipogenesis [32]. However, if metal-induced oxidative stress is a major cause of obesity, it is not clear why some metals, such as barium and thallium, positively associate with obesity, whereas other metals, such as lead, cadmium, and cesium negatively associate with obesity.

The finding that lead, cadmium, cobalt, and cesium negatively associate with obesity is surprising. A recent study has shown that lead is a predictor of diabetic neuropathy, and that chelation therapy that reduces the lead burden slows the rate of diabetic neuropathy [19]. Also, even in patients without diabetes, lead chelation therapy has been shown to slow the progression of chronic renal diseases [33,34]. Furthermore, using data from the Normative Aging Study, Tsaih and colleagues [35] showed significant interactions of blood lead and tibia lead with diabetes and renal function. For example, increasing the tibia lead level from the lowest to the highest quartiles was associated with an increase in the rate of rise of serum creatinine that was 17.6 fold greater in diabetics than in non-diabetics. The implication of this result is that lead damages the kidneys much more readily in diabetics than in non-diabetics [35]. Our study suggests that cadmium, cobalt, and cesium might function in a manner similar to lead in that, for whatever reason, they all negatively associate with obesity. It will be interesting to determine whether, like lead, any of the other toxic metals in our study positively associate with diabetic neuropathy.

However, urine lead levels, which were all that is available in the NHANES 99-02 studies analyzed here, only reflect recent lead exposures and the abovementioned studies were careful to also analyze total lead burden by measuring lead levels after EDTA chelation [19,33,34], which mobilizes lead from the bone to the circulation, or X-ray analyses of bone lead [35]. Blood lead levels are only proportional to body lead levels if there is a constant exposure to lead. However, blood lead levels have fallen over the past three decades in the U.S. because of the elimination of leaded paint and gasoline in the late 1970s, whereas the body burden of lead can last for several decades [36].

**Figure 1.**
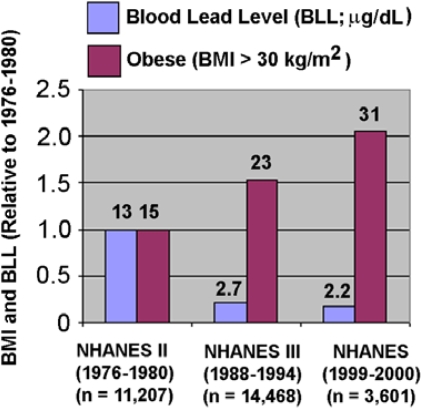
There is a negative association between blood lead levels and obesity from 1976 to 2000.

It is possible that the negative association between obesity and blood lead levels is misleading. There is a positive association between creatinine levels and obesity, and several studies have shown that body lead levels are proportional to creatinine levels because, as mentioned above, lead positively associates with kidney dysfunction and this prevents proper filtration of creatinine, which is produced from creatine kinase breakdown in muscles from the blood [19,33,34]. Also, a recent study has shown that recent severe weight loss can cause the release of stored lead from bones [37], and the weight history of the NHANES sample is not completely known. However, as shown in Figure 1, there has been a steady decrease in blood lead levels from 1977 to 2000 at the same time that there has been a steady increase in obesity in the NHANES populations. This result is consistent with lead being negatively associated with obesity. We note that Figure 1 is based on NHANES population studies, which are cross-sectional in that both metals and outcomes are assessed at the same time. Further prospective studies are needed to address the temporality of these findings.

In conclusion, there is, by and large, a negative association of heavy toxic metal urine concentrations with BMI and WC. This suggests that the association of heavy metals with BMI and WC is still unclear because the findings here are in the opposite direction of past research and hypotheses. Future research should be further conducted in order to establish a clear understanding of the association of heavy metals with BMI and WC.

## Figures and Tables

**Table 1.** Descriptive Statistics for Detectable Toxins.

	**BMI**			**Waist Circumference (WC)**		

	n	Mean	95% CI	n	Mean	95% CI

Gender						
Male	1886	25.99 (0.19)	25.60, 26.37	1889	92.45 (0.49)	91.46, 93.44
Female	1930	26.79 (0.29)	26.19, 27.39	1936	88.82 (0.70)	87.38, 90.27
Ethnicity						
Mexican American	981	26.48 (0.35)	25.77, 27.19	979	89.80 (0.78)	88.21, 91.39
Other Hispanic	206	25.74 (0.41)	24.89, 26.58	206	87.52 (0.82)	85.83, 89.20
Non-Hispanic White	1531	26.30 (0.24)	25.80, 26.79	1542	91.12 (0.60)	89.89, 92.35
Non-Hispanic Black	959	27.50 (0.30)	26.88, 28.12	957	90.50 (0.70)	88.63, 91.47
Other Race	139	26.15 (1.19)	23.72, 28.58	141	89.90 (3.11)	83.55, 96.26
Age						
06–18 yrs	1494	20.72 (0.19)	20.34, 21.11	1492	72.26 (0.52)	71.19, 73.32
19–28 yrs	491	26.85 (0.47)	25.89, 27.81	487	90.48 (0.96)	88.51, 92.45
29–39 yrs	426	27.56 (0.41)	26.72, 28.40	426	92.80 (0.93)	90.89, 94.71
40+ yrs	1405	28.44 (0.29)	27.84, 29.05	1420	98.16 (0.72)	96.68, 99.63

**Table 2.** Correlations Among all Variables in the Models.

Variables	1	2	3	4	5	6	7	8	9	10	11	12	13	14
1. BMI	1													
2. WC	0.91	1												
3. PIR	0.01	0.07	1											
4. Age	0.36	0.49	0.17	1										
5. Creatinine	0.08	0.09	−0.04	−0.14	1									
6. Barium	0.01	−0.01	−0.03	−0.16	0.48	1								
7. Cadmium	0.21	0.28	0.01	0.44	0.39	0.16	1							
8. Cobalt	−0.06	−0.09	−0.13	−0.21	0.66	0.56	0.26	1						
9. Cesium	0.01	0.01	0.02	−0.07	0.77	0.47	0.34	0.60	1					
10. Molybdenum	−0.05	−0.07	−0.05	−0.19	0.69	0.42	0.15	0.59	0.63	1				
11. Lead	−0.20	0.01	−0.13	0.04	0.63	0.44	0.38	0.52	0.59	0.47	1			
12. Antimony	−0.01	−0.02	−0.15	−0.21	0.62	0.34	0.23	0.47	0.46	0.48	0.49	1		
13. Thallium	0.02	0.01	0.01	−0.16	0.77	0.45	0.29	0.58	0.83	0.63	0.57	0.47	1	
14. Tungsten	−0.07	−0.10	−0.08	−0.25	0.49	0.33	0.04	0.45	0.38	0.51	0.35	0.42	0.38	1

**Table 3.** Regression Models for All Participants.

	BMI		WC		WC|BMI	

Variable	Beta	95% CI	Beta	95% CI	Beta	95% CI

Intercept	6.96*	0.80, 13.11	32.03*	17.80, 46.26	4.06	−1.09, 9.22
Mexican American	0.94	−0.47, 2.36	1.77	−1.77, 5.31	−0.53	−1.77, 0.71
Other Hispanic	−0.51	−2.19, 1.17	−2.58	−6.45, 1.29	−1.64	−3.00, −0.29
White	−0.71	−2.07, 0.65	−1.87	−5.42, 1.68	−0.24	−1.53, 1.05
Black	0.51	−0.94, 1.95	−2.03	−5.59, 1.52	−2.94*	−4.03, −1.85
Male	−1.07*	−1.64, −0.50	3.22*	1.79, 4.65	5.56*	4.91, 6.21
PIR	−0.48*	−0.69, −0.27	−1.17*	−1.66, −0.69	−0.09	−0.26, 0.08
Age	0.55*	0.48, 0.61	1.53*	1.39, 1.66	0.22*	0.17, 0.28
Age-2	0.00*	−0.01, −0.00	−0.01*	−0.01, −0.01	−0.00*	−0.00, −0.00
BMI					3.47*	3.21, 3.72
BMI-2					−0.02*	−0.02, −0.02
ln(Creatine)	2.61*	1.77, 3.45	7.31*	5.32, 9.31	0.73	−0.17, 1.62
ln(Barium)	0.59*	0.20, 0.99	1.51*	0.60, 2.42	0.11	−0.20, 0.42
ln(Cadmium)	−0.49*	−0.75, −0.22	−1.06*	−1.66, −0.47	0.10	−0.16, 0.35
ln(Cobalt)	−0.64*	−1.18, −0.10	−1.26	−2.54, 0.02	0.24	−0.23, 0.71
ln(Cesium)	−1.10*	−1.75, −0.46	−3.14*	−4.90, −1.38	−0.31	−0.97, 0.35
ln(Molybdenum)	−0.15	−0.50, 0.20	−0.74	−1.50, 0.01	0.29	−0.70, 0.13
ln(Lead)	−1.31*	−1.66, −0.96	−3.46*	−4.36, −2.56	−0.40	−0.82, 0.03
ln(Antimony)	0.36	−0.30, 1.03	1.12	−0.60, 2.84	0.46	−0.10, 1.03
ln(Thallium)	0.92*	0.33, 1.51	1.94*	0.51, 3.38	−0.24	−0.92, 0.45
ln(Tungsten)	0.02	−0.33, 0.37	−0.13	−0.99, 0.72	−0.02	−0.40, 0.36

Joint Test (All Metals)	<0.00005		<0.00005		0.0304	
Joint Test (Interactions)	0.0093		0.0025		<0.00005	

R-Squared (No Metals)	0.2479		0.3663		0.9006	
R-Squared (All Metals)	0.2823		0.3983		0.9013	
R-Squared (Interactions)	0.2853		0.4033		0.9037	

N	3,816		3,825		3,785	

Note.

* indicates p < 0.05. WC refers to waist circumference. Joint Test refers to the simultaneous test of all heavy metals.

**Table 4.** Least Squares Means for BMI and WC Additive Models.

	BMI			WC		

	n	Mean	95% CI	n	Mean	95% CI

Gender						
Male	1,886	25.85^a^ (0.20)	25.46, 26.24	1,889	92.24^a^ (0.50)	91.26, 93.22
Female	1,930	26.92^a^ (0.24)	26.45, 27.39	1,936	89.02^a^ (0.50)	88.04, 90.00
Ethnicity						
Mexican American	981	27.75^ab^ (0.34)	27.08, 28.42	979	93.96^abc^ (0.82)	92.35, 95.57
Other Hispanic	206	26.30^a^ (0.42)	25.48, 27.12	206	89.61^a^ (0.79)	88.06, 91.16
Non-Hispanic White	1,531	26.10^bc^ (0.21)	25.69, 26.51	1,542	90.32^b^ (0.42)	89.50, 91.14
Non-Hispanic Black	959	27.32^c^ (0.31)	26.71, 27.93	957	90.16^c^ (0.75)	88.69, 91.63
Other Race	139	26.81 (0.71)	25.42, 28.20	141	92.19 (1.74)	88.78, 95.60

Note. Numbers in parentheses are standard errors. Cell means that share the same superscript are significantly different at α = 0.05.

**Table 5.** BMI by Metal Quartiles for All Participants.

Quartile	Barium	Cadmium	Cesium	Lead	Thallium
25	25.46 (0.30)	27.38 (0.38)	26.87 (0.43)	27.50 (0.37)	26.06 (0.40)
50	26.41 (0.30)	27.12 (0.29)	26.16 (0.26)	26.93 (0.32)	26.32 (0.30)
75	26.44 (0.28)	25.99 (0.27)	26.35 (0.28)	26.26 (0.31)	26.77 (0.36)
100	27.17 (0.38)	25.34 (0.34)	26.17 (0.39)	24.67 (0.26)	26.45 (0.36)

Note. N = 3,816. Numbers in parentheses are standard errors.

**Table 6.** Waist Circumference by Metal Quartiles for All Participants.

Quartile	Barium	Cadmium	Cesium	Lead	Thallium
25	88.66 (0.75)	92.60 (0.81)	92.61 (1.03)	93.60 (0.93)	89.04 (0.76)
50	90.29 (0.62)	91.79 (0.54)	90.19 (0.69)	91.64 (0.62)	90.94. (0.70)
75	90.54 (0.66)	89.53 (0.59)	90.14 (0.69)	90.37 (0.68)	91.52 (0.84)
100	92.51 (0.81)	88.82 (0.77)	89.24 (0.91)	86.01 (0.70)	90.87 (0.86)

Note. N = 3,825. Numbers in parentheses are standard errors.

**Table 7.** Regression Models for All Participants without Creatinine as Covariate.

	BMI		WC		WC|BMI	

Variable	Beta	95% CI	Beta	95% CI	Beta	95% CI

Intercept	18.84*	15.69, 21.98	65.56*	57.92, 73.20	6.86*	3.37, 10.34
Mexican American	0.94	−0.58, 2.45	1.80	−1.92, 5.52	−0.54	−1.78, 0.69
Other Hispanic	−0.34	−2.12, 1.44	−2.05	−6.05, 1.95	−1.60	−2.92, −0.28
White	−0.69	−2.18, 0.79	−1.80	−5.65, 2.05	−0.23	−1.52, 1.06
Black	0.99	−0.58, 2.56	−0.64	−4.53, 3.26	−2.81*	−3.91, −1.71
Male	−0.59*	−1.17, −0.00	4.57*	3.08, 6.05	5.69*	5.10, 6.28
PIR	−0.47*	−0.70, −0.25	−1.15*	−1.67, −0.63	−0.08	−0.25, 0.09
Age	0.58*	0.52, 0.65	1.63*	1.49, 1.76	0.23*	0.18, 0.28
Age-2	−0.01*	−0.01, −0.00	−0.01*	−0.01, −0.01	−0.00*	−0.00, −0.00
BMI	.	.	.	.	3.51*	3.26, 3.76
BMI-2	.	.	.	.	−0.02*	−0.03, −0.02
ln(Barium)	0.60*	0.20, 1.00	1.53*	0.62, 2.44	0.11	−0.20, 0.41
ln(Cadmium)	−0.33*	−0.59, −0.06	−0.60	−1.21, 0.01	0.14	−0.10, 0.39
ln(Cobalt)	−0.30	−0.80, 0.20	−0.31	−1.49, 0.88	0.34	−0.10, 0.78
ln(Cesium)	−0.52	−1.05, 0.01	−1.51*	−2.79, −.22	−0.14	−0.78, 0.49
ln(Molybdenum)	0.21	−0.18, 0.61	0.25	−0.58, 1.08	−0.19	−0.59, 0.21
ln(Lead)	−1.22*	−1.60, −0.84	−3.21*	−4.26, −2.15	−0.36	−0.79, 0.07
ln(Antimony)	0.87*	0.25, 1.48	2.53*	0.89, 4.17	0.60*	0.10, 1.09
ln(Thallium)	1.43*	0.83, 2.03	3.40*	1.94, 4.86	−0.11	0.77, 0.55
ln(Tungsten)	0.15	−0.18, 0.48	0.23	−0.06, 1.06	0.02	−0.35, 0.38

Joint Test (All Metals)	<0.00005		<0.00005		0.0827	
Joint Test (Interactions)	0.0145		0.0004		<0.00005	

R-Squared (No Metals)	0.2364		0.3554		0.9005	
R-Squared (All Metals)	0.2664		0.3806		0.9011	
R-Squared (Interactions)	0.2707		0.3872		0.9036	

N	3,816		3,825		3,785	

Note.

* indicates p < 0.05. WC refers to waist circumference. Joint Test refers to the simultaneous test of all heavy metals.

**Table 8.** Regression for Obesity and Lead Controlling for Model Descriptives.

	BMI		WC	

Variable	Beta	p-value	Beta	p-value

Intercept	16.41	<0.00005	57.08	<0.00005
Mexican American	0.69	0.4688	1.09	0.6339
Other Hispanic	−0.44	0.7006	−2.45	0.3624
White	−0.80	0.3817	−2.16	0.3593
Black	1.11	0.2494	−0.34	0.8842
Male	−0.54	0.0752	4.53	<0.00005
Age	0.50	<0.00005	1.45	<0.00005
Age-2	−0.00	<0.00005	−0.01	<0.00005
ln(Lead)	−0.22	0.1072	−0.61	0.0506

R-Squared	0.2292		0.3491	

N	3,816		3,825	

Note. WC refers to waist circumference. Model is for all participants.

**Table 9.** Regression Models for Adolescents.

	BMI		WC		WC|BMI	

Variable	Beta	95% CI	Beta	95% CI	Beta	95% CI

Intercept	7.81*	0.87, 14.76	19.93*	1.50, 38.36	−5.62	−13.10, 1.86
Mexican American	0.83	−0.30, 1.97	2.67	−0.27, 5.62	0.86	−0.83, 2.55
Other Hispanic	0.12	−1.58, 1.81	−0.49	−4.48, 3.50	−0.71	−2.31, 0.89
White	−0.87	−1.85, 0.11	−1.55	−4.18, 1.08	0.61	−0.81, 2.02
Black	0.53	−0.55, 1.61	−1.61	−4.34, 1.11	−2.64*	−4.05, −1.23
Male	−0.71*	−1.24, −0.18	−0.92	−2.51, 0.66	0.94*	0.21, 1.66
PIR	−0.37*	−0.60, −0.15	−0.86*	−1.48, −0.24	0.10	−0.15, 0.35
Age	1.14*	0.54, 1.73	5.58*	4.07, 7.08	2.56*	2.13, 2.99
Age−2	−0.02*	−0.05, 0.00	−0.15*	−0.21, −0.08	−0.08*	−0.10, −0.06
BMI					3.10*	2.75, 3.44
BMI−2					−0.01*	−0.02, −0.01
ln(Creatine)	1.09	−0.22, 2.39	2.86	−0.62, 6.35	0.25	−0.73, 1.22
ln(Barium)	0.51*	0.12, 0.90	1.07*	0.01, 2.13	−0.09	−0.41, 0.23
ln(Cadmium)	−0.22	−0.45, 0.01	−0.66*	−1.30, −0.03	−0.12	−0.27, 0.02
ln(Cobalt)	−0.78*	−1.34, −0.22	−2.07*	−3.65, −0.50	−0.06	−0.58, 0.46
ln(Cesium)	−0.31	−0.69, 0.07	−0.50	−1.63, 0.64	0.38	−0.38, 1.14
ln(Molybdenum)	−0.12	−0.67, 0.43	−0.42	−1.83, 0.98	−0.18	−0.62, 0.26
ln(Lead)	−0.81*	−1.30,	−2.22*	−3.73, −0.71	−0.26	−0.83, 0.31
ln(Antimony)	0.29	−0.311	0.80	−0.80, 2.40	0.11	−0.48, 0.71
ln(Thallium)	0.47	−0.34, 0.92	1.21	−0.54, 2.96	−0.03	−0.85, 0.80
ln(Tungsten)	0.02	−0.17, 1.11	0.08	−1.04, 1.20	−0.00	−0.31, 0.31
		−0.43, 0.46				

N	1,494		1,492		1,487	

Note.

* indicates p < 0.05.

**Table 10.** BMI by Metal Quartiles for Adolescents.

Quartile	Barium	Cobalt	Lead
25	19.86 (0.26)	21.48 (0.46)	21.21 (0.32)
50	20.55 (0.28)	21.05 (0.32)	21.01 (0.37)
75	20.93 (0.30)	20.41 (0.28)	20.95 (0.33)
100	21.23 (0.34)	19.93 (0.33)	19.34 (0.31)

Note. N = 1,494. Numbers in parentheses are standard errors.

**Table 11.** Waist Circumference by Metal Quartiles for Adolescents.

Quartile	Barium	Cobalt	Lead
25	70.66 (0.72)	74.06 (1.10)	73.67 (1.01)
50	71.94 (0.70)	72.98 (0.90)	72.50 (0.87)
75	72.64 (0.78)	71.74 (0.86)	73.01 (0.79)
100	73.17 (1.03)	70.16 (0.88)	68.81 (0.86)

Note. N = 1,492. Numbers in parentheses are standard errors.

**Table 12.** Regression Models for Adults.

	BMI		WC		WC|BMI	

Variable	Beta	95% CI	Beta	95% CI	Beta	95% CI

Intercept	8.57*	0.21, 16.93	49.23*	30.40, 68.07	16.91*	9.75, 24.07
Mexican American	0.79	−1.23, 2.81	0.84	−3.85, 5.53	−1.11	−2.62, 0.41
Other Hispanic	−0.77	−3.09, 1.56	−2.96	−8.29, 2.36	−1.61	−3.33, 0.11
White	−0.70	−2.63, 1.22	−1.97	−6.92, 2.99	−0.45	−2.02, 1.12
Black	0.52	−1.54, 2.57	−1.60	−6.66, 3.47	−2.70*	−4.20, −1.21
Male	−1.27*	−1.99, −0.54	4.53*	2.57, 6.48	7.20*	6.18, 8.22
PIR	−0.45*	−0.71, −0.20	−1.05*	−1.63, −0.47	−0.09	−0.30, 0.11
Age	0.47*	0.32, 0.62	1.14*	0.81, 1.47	0.10*	0.01, 0.18
Age-2	−0.00*	−0.01, −0.00	−0.01*	−0.01, −0.01	0.00	−0.00, 0.00
BMI	.	.	.	.	3.25*	2.83, 3.66
BMI-2	.	.	.	.	−0.02*	−0.02, −0.01
ln(Creatine)	2.69*	1.55, 3.83	5.44*	2.72, 8.17	−0.84	−1.92, 0.25
ln(Barium)	0.58*	0.07, 1.09	1.47*	0.28, 2.66	0.12	−0.26, 0.49
ln(Cadmium)	−0.86*	−1.29, −0.44	−1.54*	−2.57, −0.51	0.40	−0.11, 0.91
ln(Cobalt)	−0.51	−1.14, 0.13	−0.72	−2.13, 0.68	0.39	−0.24, 1.02
ln(Cesium)	−1.15*	−1.93, −0.36	−2.78*	−4.68, −0.87	0.03	−0.65, 0.71
ln(Molybdenum)	−0.13	−0.55, 0.28	−0.39	−1.34, 0.55	−0.03	−0.55, 0.49
ln(Lead)	−1.20*	−1.75, −0.65	−2.92*	−4.16, −1.67	−0.22	−0.75, 0.31
ln(Antimony)	0.41	−0.41, 1.23	1.40	−0.67, 3.47	0.64*	0.04, 1.24
ln(Thallium)	1.07*	0.38, 1.75	2.30*	0.84, 3.76	−0.23	−1.00, 0.53
ln(Tungsten)	0.08	−0.30, 0.46	0.10	−0.93, 1.14	0.07	−0.40, 0.53
						
N	2,322		2,333		2,298	

Note.

* indicates p < 0.05.

**Table 13.** BMI by Metal Quartiles for Adults.

Quartile	Barium	Cadmium	Cesium	Lead	Thallium
25	27.07 (0.38)	28.63 (0.42)	28.79 (0.52)	29.15 (0.52)	26.82 (0.48)
50	27.54 (0.36)	28.39 (0.44)	27.69 (0.35)	28.53 (0.42)	28.23 (0.46)
75	28.17 (0.36)	27.70 (0.32)	27.75 (0.35)	27.69 (0.35)	28.08 (0.49)
100	28.80 (0.44)	26.88 (0.41)	27.56 (0.45)	26.27 (0.42)	28.60 (0.38)

Note. N = 2,322. Numbers in parentheses are standard errors.

**Table 14.** Waist Circumference by Metal Quartiles for Adults.

Quartile	Barium	Cadmium	Cesium	Lead	Thallium
25	93.84 (0.95)	96.60 (0.89)	97.65 (1.25)	98.27 (1.16)	92.25 (1.01)
50	94.44 (0.73)	95.91 (0.95)	95.49 (0.80)	96.72 (0.78)	96.56 (1.07)
75	95.60 (0.78)	95.44 (0.73)	94.91 (0.80)	95.31 (0.77)	95.91 (1.16)
100	97.78 (0.93)	93.95 (1.07)	94.17 (1.07)	91.42 (1.06)	97.28 (0.85)

Note. N = 2,333. Numbers in parentheses are standard errors.
